# *Hibiscus sabdariffa* Leaf Extract Inhibits Human Prostate Cancer Cell Invasion via Down-Regulation of Akt/NF-κB/MMP-9 Pathway

**DOI:** 10.3390/nu7075065

**Published:** 2015-06-24

**Authors:** Chun-Tang Chiu, Jing-Hsien Chen, Fen-Pi Chou, Hui-Hsuan Lin

**Affiliations:** 1 Institute of Biochemistry and Biotechnology, Chung Shan Medical University, No. 110, Sec. 1, Jianguo N. Road, Taichung City 40201, Taiwan; E-Mail: nydrskin@yahoo.com.tw; 2 School of Nutrition, Chung Shan Medical University, No. 110, Sec. 1, Jianguo N. Road, Taichung City 40201, Taiwan; E-Mail: cjh0828@csmu.edu.tw; 3 School of Medical Laboratory and Biotechnology, Chung Shan Medical University, No. 110, Sec. 1, Jianguo N. Road, Taichung City 40201, Taiwan; 4 Department of Clinical Laboratory, Chung Shan Medical University Hospital, No. 110, Sec. 1, Jianguo N. Road, Taichung City 40201, Taiwan

**Keywords:** *Hibiscus sabdariffa* leaf, metastasis, polyphenols, human prostate cancer, Akt/NF-κB/MMP-9 cascade pathway

## Abstract

*Hibiscus sabdariffa* leaf has been previously shown to possess hypoglycemic, hypolipidemic, and antioxidant effects, and induce tumor cell apoptosis. However, the molecular mechanisms involved in the anticancer activity of *H. sabdariffa* leaf extract (HLE) are poorly understood. The object of the study was to examine the anti-invasive potential of HLE. First, HLE was demonstrated to be rich in polyphenols. The results of wound-healing assay and *in vitro* transwell assay revealed that HLE dose-dependently inhibited the migration and invasion of human prostate cancer LNCaP (lymph node carcinoma of the prostate) cells under non-cytotoxic concentrations. Our results further showed that HLE exerted an inhibitory effect on the activity and expressions of matrix metalloproteinase-9 (MMP-9). The HLE-inhibited MMP-9 expression appeared to be a consequence of nuclear factor-kappaB (NF-κB) inactivation because its DNA-binding activity was suppressed by HLE. Molecular data showed all these influences of HLE might be mediated via inhibition of protein kinase B (PKB, also known as Akt)/NF-κB/MMP-9 cascade pathway, as demonstrated by the transfection of *Akt1* overexpression vector. Finally, the inhibitory effect of HLE was proven by its inhibition on the growth of LNCaP cells and the expressions of metastasis-related molecular proteins *in vivo*. These findings suggested that the inhibition of MMP-9 expression by HLE may act through the suppression of the Akt/NF-κB signaling pathway, which in turn led to the reduced invasiveness of the cancer cells.

## 1. Introduction

Prostate cancer (CaP) is a very common male-specific malignancy, and the second most common cancer among men in the world [1]. Thus, developing novel treatment options for CaP has become an important medical need. Since CaP is so highly sensitive to androgens, the intrinsic androgenic, glucocorticoid, and estrogenic-like activities of nutri-medicinal plants or herbs have potential for use in the treatment of CaP [2]. In recent years, many anticancer agents appeared to target signaling intermediates in metastatic pathways. Current reports revealed that the inhibition of metastasis in CaP cells by tea polyphenols [3] and curcumin [4] was related to the signal transduction regulation.

Tumor metastasis occurs by a series of steps, including vessel formation, cell attachment, invasion, and cell proliferation, and is regulated by extremely complicated mechanisms [5]. The degradation of basement membranes and the stromal extracellular matrix (ECM) are crucial steps for tumor invasion and metastasis. The matrix metalloproteinases (MMPs) family of human zinc-dependent endopeptidases is responsible for the degradation of the ECM [6]. Among them, gelatinases (MMP-2 and MMP-9) efficiently degrade native collagen types IV and V, fibronectin, and elastin. The expression of the MMPs gene is primarily regulated at the transcriptional (through activator protein-1 (AP-1) or nuclear factor-kappaB (NF-κB) via mitogen-activated protein kinase (MAPK) or phosphatidylinositol 3-kinase (PI3K)/protein kinase B (PKB, also known as Akt) pathways) and posttranscriptional levels, and at the protein level via their activators or inhibitors, and their cell surface localization [6,7,8]. MMPs and their regulatory pathways have been considered promising targets for anticancer drugs and chemotherapeutic agents [9].

*Hibiscus sabdariffa* Linne (*Malvaceae*, local name Roselle), an attractive plant believed to be native to Africa, is cultivated in both Sudan and Eastern Taiwan [10]. Typically, the calyces of the plant are used in the manufacturing of beverages, jam, and vegetable gelatin [11]. However, *H. sabdariffa* L. has many other applications in Africa. Among the nourishing applications, the leaves are used like vegetables in the preparation of soups and sauces [11]. Moreover, many medicinal applications of this plant have been developed around the world. In folk herbal medicine, it is used to treat hypertension [12,13], pyrexia [12], and liver disorders [14,15], and is used for its immunemodulating effect [16] in Asia. An aqueous extract of dried flowers of *H. sabdariffa* L. has been used as an effective treatment against leukemia and gastric carcinoma, due to its high content in polyphenols [17,18]. Previous studies have demonstrated that leaves of *H. sabdariffa* possess hypoglycemic [19], hypolipidemic [20,21], antioxidant [21,22], and estrogenic-like effects [23]. Recent studies suggested that *H. sabdariffa* is an interesting nutri-medicinal plant with multiple pharmacological activities [24,25,26], and *H. sabdariffa* leaf extract (HLE) has the potential to be developed as a chemotherapeutic agent. In anticancer studies, HLE-induced apoptosis through mediated intrinsic and extrinsic apoptotic pathways in human prostate cancer cells [27]. However, the molecular mechanisms involved in the anticancer activity of HLE are poorly understood. The object of the study was to examine the anti-invasive potential of HLE.

Previous studies on functions of HLE have been mainly focused on its antioxidant and apoptosis-inducing activities, whereas the effect of HLE on metastasis and invasion of tumor cells has not been clearly clarified. Since cancer metastasis and invasion are highly related to the degradation of the ECM, intercellular adhesion, and cellular motility, this study explored the effects of HLE on MMPs expression, as well as the activities of Akt, MAPK, and transcriptional factors (AP-1 and NF-κB) on LNCaP (lymph node carcinoma of the prostate) cells, an androgen-responsive human CaP cell line, to explore the underlying mechanism for the action of HLE in cancer cell invasion *in vitro*. Additionally, the effect of HLE was shown by its inhibition of the growth of LNCaP cells in xenograft tumor studies. The detailed signaling pathway involved in HLE-inhibited CaP metastasis *in vivo* is also included.

## 2. Experimental Section

### 2.1. Preparation of H. Sabdariffa Leaf Extract and Functional Components Assay

HLE was prepared from *H. sabdariffa* (Malvaceae) leaves. The dried leaves of *H. sabdariffa* L. (100 g) were macerated with hot water (95 °C, 4000 mL) for 2 h and the aqueous extract was evaporated under vacuum at −85 °C. The extracted solution was filtered, and then lyophilized to obtain approximately 26.6 g of HLE and stored at −20 °C before use.

The functional components of HLE were determined as follows. The concentration of total polyphenol was analyzed according to the Folin-Ciocalteau method. HLE (0.1 mg) was first dissolved in a test tube with 1 mL of distilled water, and then Folin-Ciocalteau reagent (2N, 0.5 mL) were added and mixed in thoroughly. After an interval of 3 min, 3 mL of 2% Na_2_CO_3_ solution was added, and the mixture was allowed to stand for 15 min with intermittent mixing. The absorbance of the mixture at 750 nm was measured on a Hitachi U-3210 spectrophotometer (Hitachi, Tokyo, Japan). A standard curve using gallic acid (GA) was also prepared. Total flavonoid content was determined by the Jia method using rutin as a standard. Next, 0.5 mL of the HLE (1 mg/mL) was diluted with 1.25 mL of distilled water. Then, 75 μL of a 5% NaNO_2_ solution were added to the mixture. After 6 min, 150 μL of a 10% AlCl_3_·6H_2_O solution was added, and the mixture was allowed to stand for another 5 min. Afterwards, 0.5 mL of 1 M NaOH and 2.5 mL of distilled water were added. The solution was mixed, and the absorbance was measured immediately against the prepared control at 510 nm. Total anthocyanin content in HLE was determined using the Fuleki and Francis method. In addition, 10 mL of HLE (1 mg/mL) were diluted to 50 mL with pH 1.0 and 4.5 buffer, respectively. The absorbance of the samples was measured at 535 nm, using distilled water as a control. The difference in absorbance was obtained by subtracting the total absorbance at pH 4.5 from the total absorbance at pH 1.0. Both of these values were calculated from the absorbance readings using the appropriate dilution and calculation factors. The functional components content of the final extract HLE are summarized in Table 1.

**Table 1:** Composition of the *Hibiscus sabdariffa* leaf extract (HLE).

Polyphenolic compound	Peak no. ^a^	HLE (%)
Catechin	3	5.3 ± 1.5
Ellagic acid	6	33.6 ± 6.0
EGC	8	0.9 ± 0.8
Total polyphenol (Folin-Ciocalteu method)		5.2 ± 0.1
Total flavonoid (Jia method)		21.0 ± 1.7
Total anthocyanin (Fuleki and Francis method)		1.9 ± 1.2

^a^ Phenolic compounds correspond to peaks as in high performance liquid chromatography (HPLC) chromatogram of 10 kinds of standard polyphenols.

### 2.2. High Performance Liquid Chromatography (HPLC) Assay for HLE

The components of HLE were determined by HPLC analysis using a Hewlett-Packard Vectra 436/33N system (Waldbronn, Germany) with a diode array detector. The HPLC method employed a 5 μm RP-18 column (4.6 × 150 mm i.d.). The HLE were filtered through a 0.45 μm filter disc, and 20 μm were injected onto the column. The chromatography was monitored at 285 and 345 nm, and UV spectra were collected to confirm peak purity. The mobile phase contained two solvents (A, formic acid/water = 10:90; B, formic acid/water/acetonitrile = 10:60:30) run by a linear gradient method at room temperature as follows: from 10% B to 40% B (flow rate = 1.0 mL/min) over 25 min.

### 2.3. Cell Culture and Treatment

Human CaP LNCaP cells were obtained from American Type Culture Collection (ATCC, Manassas, VA, USA). The cells were cultured in growth media prepared according to ATCC recommendations, and supplemented with 10% fetal bovine serum (FBS), 2 mM glutamine, and 100 U/mL penicillin-streptomycin mixed antibiotics. All cell cultures were maintained at 37 °C in a humidified atmosphere of 5% CO_2_. Cells were seeded at a density of 10^6^ onto each 10 cm Petri dish 24 h before HLE treatment.

### 2.4. Assessment of Cell Viability

Cells were seeded at a density of 10^5^ cells/mL and incubated with HLE at various concentrations (0–20 mg/mL) for 24, 48, and 72 h. Afterwards, the medium was changed and 3-(4,5-dimethylthiazol-2-yl)-2,5-diphenyltetrazolium bromide (MTT, 0.1 mg/mL) was added for 4 h. The viable cell number was directly proportional to the production of formazan which, following solubilization with isopropanol, was measured spectrophotometrically at 563 nm. The MTT assay was used to evaluate the effect of the test drugs on cell viability, as described previously [28], and to determine the non-cytotoxic concentrations.

### 2.5. Wound-Healing Assay

LNCaP cells were grown to confluent monolayer in six-well petri dishes for 24 h in serum-free medium (three dishes per group). According to the MTT results, to provide a maximum dynamic range for quantifying non-cytotoxic responses, treatment with HLE at various concentrations (0, 0.1, 0.25, and 0.5 mg/mL) was chosen in the subsequent experiments. The medium was replaced with serum-containing medium following the treatments of HLE, and the monolayers were disrupted (*i.e.*, wounded) by scraping them with a P200 micropipette tip. At the indicated times (0, 24, 48, and 72 h) after scraping, the cells were washed twice in phosphate buffered saline (PBS, pH 7.4). The number of cells in the denuded (scraped) zone of each dish was counted at ×100 magnification in a blinded fashion. Each dish was counted three times to ensure accuracy [29].

### 2.6. Cell Invasion Assay

LNCaP cells were incubated with HLE at various concentrations (0, 0.1, 0.25, and 0.5 mg/mL) for an additional 24 h. Afterwards, the cells were removed by trypsinizing, and their *in vitro* invasiveness was tested by the Boyden chamber invasion assay [30]. Matrigel (Collaborative Biomedical Products, Bedford, MA, USA) was diluted to 25 mg/50 mL with cold-filtered distilled water, and applied to 8-μm pore-size polycarbonate membrane filters. The above treated cells were seeded to the Boyden chamber (Neuro Probe, Cabin John, MD, USA) at the upper part, at a density of 3 × 10^5^ cells/mL in 50 μL of serum-free medium, and the bottom chamber also contained standard medium with 10% FBS. An 8-μm pore-size polycarbonate membrane filter was placed in between the upper and bottom part. After the chamber was incubated for 6 h at 37 °C, the cells that had invaded to the lower surface of the membrane were fixed with methanol and stained with Giemsa. Cell numbers in randomly selected fields were counted under a light microscope at ×400 magnification.

### 2.7. Gelatin Zymography

The activities of MMP-2 and MMP-9 in the conditioned medium were measured by gelatin-zymogram protease assays as described previously [31]. In general, samples were prepared with standard sodium dodecyl sulfate (SDS) gel-loading buffer containing 0.01% SDS, but not β-mercaptoethanol. Samples were not boiled before loading. The prepared samples (20 μg total protein) were then subjected to electrophoresis on 8% SDS polyacrylamide gels (0.75 mm thick, acrylamide/bis-acrylamide = 30/1.2) containing 0.1% gelatin. After electrophoresis, gels were washed twice with 100 mL distilled water containing 2% Triton X-100 on a gyratory shaker for 30 min at room temperature to remove SDS, and then incubated in 100 mL reaction buffer (40 mM Tris-HCl, pH 8.0, 10 mM CaCl_2_, 0.02% NaN_3_) for 12 h at 37 °C. The gels were stained with Coomassie brilliant blue R-250, followed by de-staining with methanol-acetic acid-water (50/75/875, v/v/v).

### 2.8. Western Blot Analysis

Western blotting was performed according to a previously described method and the basic methodology for the preparation of cytosolic and nuclear fractions of the cells was performed as described previously [31,32]. Antibodies against MMP-2, MMP-9, c-Jun, c-Fos, NF-κB, IκBα (inhibitor of NF-κB type α), PI3K, p-Akt, Akt, p-ERK (extracellular signal-regulated kinase)1/2, ERK1/2, and β-actin were purchased from Santa Cruz Biotechnology (Santa Cruz, CA, USA). Immunodetection was performed using an enhanced chemiluminescence detection kit.

### 2.9. Real-Time QuantitativeReverse Transcription Polymerase Chain Reaction (Real-Time qRT-PCR)

Total RNA was isolated from cells with a guanidinium chloride procedure as described previously, and the mRNA levels were analyzed by real-time qRT-PCR using a Bio-Rad iCycler system (Bio-Rad, Hercules, CA, USA) [33]. The mRNAs were reverse-transcribed into cDNAs by using an iScript cDNA synthesis kit (Bio-Rad). The specificity of primers was tested by running a regular PCR for 40 cycles at 95 °C for 20 s and 60 °C for 1 min, followed by electrophoresis on an agarose gel. The real-time PCR was performed using a SYBR supermix kit (Bio-Rad) and run for 40 cycles at 95 °C for 20 s and 60 °C for 1 min. Each 20 μL PCR mixture contained cDNA template, SYBR supermix kit, and 0.5 μM of each gene-specific primer. Specific primers were designed using Beacon Designer 2.0 software. The lengths of all amplified products are between 75 and 150 bp. The sequences of each gene-specific primer are as follows: 5′-CTGACCCCCAGTCCTATCTGCC-3′ (MMP-2 forward) and 5′-TGTTGGGAACGCCTGACTTCAG-3′ (MMP-2 reverse); and 5′-CTTTGACAGCGACAAGAAGTGG-3′ (MMP-9 forward) and 5′-GGCACTGAGGAATGATCTAAGC-3′ (MMP-9 reverse). The sequences for the house-keeping gene, β-actin, are as follows: 5′-CTGGAACGGTGAAGGTGACA-3′ (β-actin forward) and 5′-AAGGGACTTCCTGTAACAATGCA-3′ (β-actin reverse). The PCR efficiency was examined by serial dilution of the cDNA, and the PCR specificity was checked by melting curve data. Each cDNA sample was triplicated and the corresponding no-RT mRNA sample was included as a negative control. The β-actin primers were included in every plate to avoid sample variations. The mRNA level of each sample for each gene was normalized to that of the β-actin mRNA.

### 2.10. Electrophoretic Mobility Shift Assay (EMSA)

The DNA-binding activities of AP-1 and NF-κB in nuclear extracts were assessed by EMSA [34] using the Lightshift kit from Pierce (Rockford, IL, USA) with biotin-labeled, double-stranded AP-1 or NF-κB oligonucleotides (Promega, Madison, WI, USA). The binding reactions containing 10 μg of nuclear protein, 10 mM Tris, 50 mM KCl, 1 mM DTT, 5 mM MgCl_2_, 2 μg poly (dI·dC), and 2 pmol of oligonucleotide probes were incubated at room temperature for 20 min. Specific binding was confirmed by a 200-fold excess of unlabeled probes as a specific competitor. Protein DNA complexes were separated on a 6% non-denaturing acrylamide gel, and then transferred to positively charged nylon membranes and cross-linked in a Stratagene cross-linker. Band shifts were visualized with a streptavidin-horseradish peroxidase, followed by chemiluminescent detection.

### 2.11. Transient Transfection

Transient transfection assay was carried out as previously described [35]. Liposome-mediated transfection was performed using Lipofectamine^TM^ (Invitrogen, Carlsbad, CA, USA) on LNCaP cells with a control pUSEamp empty vector, as well as an expression vector for *Akt1* cDNA in pUSEamp (activated) (Upstate Biotechnology, Lake Placid, NY, USA). Briefly, LNCaP cells were plated onto six well plates overnight and transfections were carried out on cells at 70%–80% confluence the next day, using Lipofectamine reagent according to the manufacturer’s instructions. Briefly, lipofectamine (5 μL) and DNA (2 μg) were diluted in 100 μL of Roswell Park Memorial Institute (RPMI) 1640 medium followed by equilibration at room temperature for 5–10 min after mixing. The lipofectamine-DNA complex was added to LNCaP cells and incubated for 12 h. Cells were then washed with PBS and replenished with RPMI 1640 medium containing 20% serum. At 12 h after transfection, the cells were incubated with 0.5 mg/mL HLE for 24 h and then expanded for further studies.

### 2.12. Xenograft Tumor Studies

Eight-week-old athymic nude mice (18–26 g) were obtained from the National Laboratory Animal Center (Taiwan), housed in cages, and maintained at a temperature of 22 °C ± 2 °C and humidity of 65% ± 5% in a controlled animal facility with a 12 h light-dark cycle. They were allowed *ad libitum* access to water. First, 2 × 10^7^ LNCaP cells in 100 μL Matrigel were implanted into the right flank of nude mice, resulting in tumor formation. Mice were then randomly divided into two groups (12 mice per group), and 50 mg/mL HLE solution (equivalent to 1% HLE) was added to the basal diets (5 g) prepared daily for 42 days. Immediately after sacrifice, the tumor xenografts were dissected for final volume and wet weight measurement. The histological evaluation was performed as described previously [27,36]. In addition, Western blotting was carried out with tumor tissue extracts from the mice.

### 2.13. Statistical Analysis

Data are reported as means ± standard deviation (SD) of three independent experiments and evaluated by one-way analysis of variance (ANOVA) with a *post-hoc* Dunnett’s test for multi-comparison. Significant differences were established at *p* < 0.05.

## 3. Results

### 3.1. Composition of HLE

**Figure 1:**
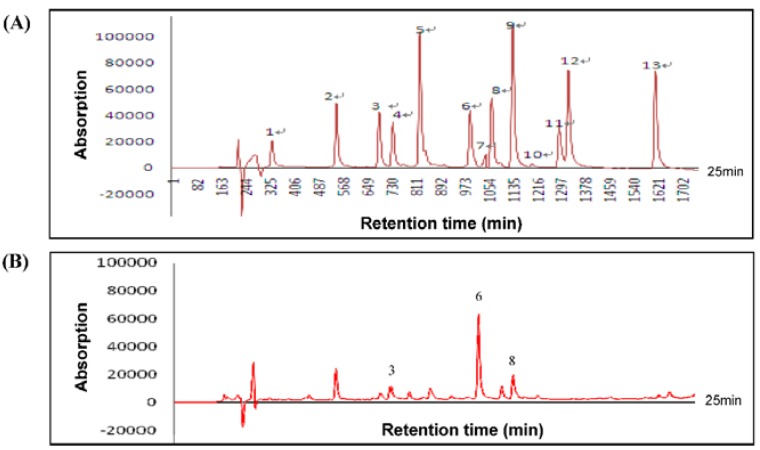
(**A**) HPLC chromatogram of 13 kinds of standard polyphenols (10 mg/mL; 10 μL). Peaks: 1, gallic acid (GA); 2, protocatechuic acid (PCA); 3, catechin; 4, procyaninidin B2; 5, (-)-epicatechin gallate (ECG); 6, ellagic acid (EA); 7, rutin; 8, (-)-epigallocatechin (EGC); 9, ferulic acid (FA); 10, gossypetin; 11, gossypin; 12, quercetin; and 13, naringenin. (**B**) HPLC chromatograms of free polyphenols from *Hibiscus sabdariffa* leaf extract (HLE) (5 mg/mL; 10 μL). Phenolic compounds correspond to peaks 3, 6, and 8 as in (**A**). Detector was set at 285 and 345 nm.

The dried leaves of *H. sabdariffa* L. (100 g) were extracted as described in Materials and Methods to produce approximately 26.6 g of a water-soluble dry powder (HLE). To further characterize composition information, HLE was also subjected to compositional determination of phenolic compounds by the HPLC technique. The HPLC analysis of 13 kinds of standard polyphenols showed the retention times of GA, protocatechuic acid (PCA), catechin, procyaninidin B2, (-)-epicatechin gallate (ECG), ellagic acid (EA), rutin, (-)-epigallocatechin (EGC), ferulic acid (FA), gossypetin, gossypin, quercetin, and naringenin: 4.6, 7.5, 9.4, 9.9, 11.2, 13.3, 14.0, 14.4, 15.3, 17.2, 17.6, 21.6, and 24.5 min, respectively (Figure 1A). According to HPLC analysis, EA (33.6% ± 6.0%) was identified to be present in the highest level in HLE, followed by catechin (5.3% ± 1.5%), and only traces of EGC (0.9% ± 0.8%) were detected (Figure 1B). The composition of the HLE used for all of the experiments in this paper is shown in Table 1. The data confirmed that polyphenols were indeed present in HLE.

### 3.2. Effects of HLE on the Motility and Invasive Ability of LNCaP Cells

**Figure 2:**
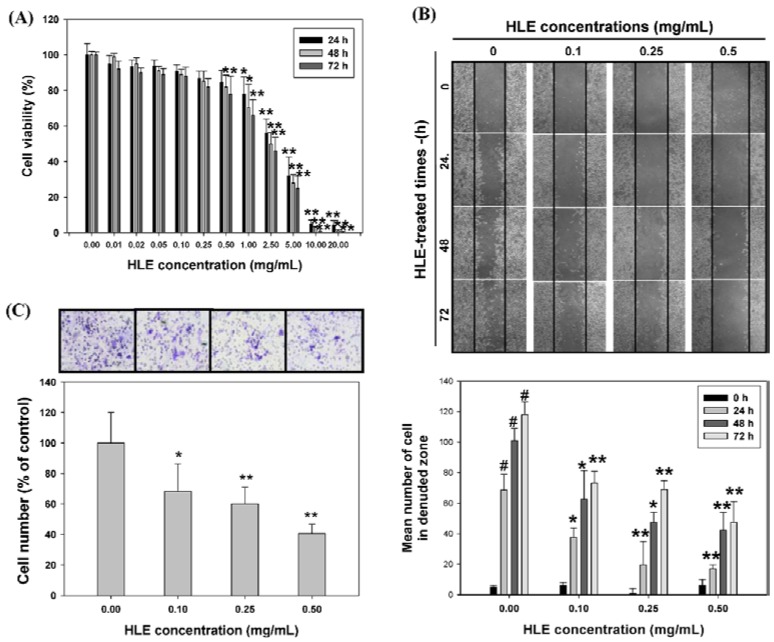
Effects of *Hibiscus sabdariffa* leaf extract (HLE) on cell viability, wound-healing ability, and invasion of LNCaP cells. (**A**) LNCaP cells were treated with or without HLE under different concentrations (0–20.0 mg/mL) for 24, 48, and 72 h. Cell viability was analyzed by MTT assay. The result represents the average of three independent experiments ±SD. * *p* < 0.05, ** *p* < 0.01 compared with the respective time point of the control group. (**B**) Monolayers of growth-arrested LNCaP cells treated with HLE (0, 0.1, 0.25, and 0.5 mg/mL) were scraped and the number of cells in the denuded zone (*i.e.*, wound) was quantified after 24, 48, and 72 h under light microscopy. Quantitative assessment of the mean number of cells in the denuded zone represents the average of three independent experiments ±SD. # *p* < 0.01 compared with the 0 h. * *p* < 0.05, ** *p* < 0.01 compared with the respective time point of the control group. (**C**) LNCaP cells were pre-treated with HLE (0, 0.1, 0.25, and 0.5 mg/mL) for 24 h. Treated cells were then subjected to analyses for invasion as described in Materials and Methods. Representative photomicrographs of the membrane-associated cells were assayed by Giemsa stain. The purple parts indicated the membrane-associated cells. “% of control” denotes the mean number of cells in the membrane expressed as a proportion of that control group and the average of three independent experiments ±SD. * *p* < 0.05, ** *p* < 0.01 compared with the control.

HLE has been reported to be a chemotherapeutic agent as evidenced by its ability to induce tumor cell apoptosis. In this study, we first determined the cytotoxicity of HLE by treating LNCaP cells (10^5^ cells/mL) with HLE at various concentrations (0.01–20 mg/mL) for 24, 48, and 72 h, followed by MTT assay. Compared to that of the control (untreated group), the cell viability was decreased by HLE at dosages above 0.5 mg/mL in a time- and dose-dependent manner, but was not significantly affected at 0.5 mg/mL of HLE for 24 h. The 50% growth inhibition value (GI_50_) of HLE was about 3.0 mg/mL for the 24 h incubation (Figure 2A). In addition, the results confirmed that the culture mediums of LNCaP cells following treatment with 0.05–0.5 mg/mL of HLE, including approximately 50–500 μM of EA, have little pH changes (pH 7.54–7.63) (Figure S1). Therefore, the results demonstrated that the 24-h treatments of HLE at lower doses ranging from 0.1 to 0.5 mg/mL have little cytotoxicity to LNCaP. This concentration range was then applied in all subsequent experiments.

The effect of HLE on LNCaP cell migration was determined by the wound-healing assay in which cells were diminished to migrate by the physical wounding of cells plated on fibronectin pre-coated plates. As shown in Figure 2B (upper panel), the untreated cells for 24, 48, and 72 h, an apparent and gradual increase of cells in the denuded zone was observed under light microscopy. LNCaP cells exposed to 0.1, 0.25, and 0.5 mg/mL of HLE showed reduced ability to migrate and fill the wounded area as compared to the control cells. The quantitative data in Figure 2B (bottom panel) revealed that HLE could inhibit the migration of LNCaP cells in a dose- and time-dependent manner. To further examine the effect of HLE on the invasive ability of LNCaP cells, a Boyden chamber coated with Matrigel was used in a dosage experiment. LNCaP cells treated with HLE (0, 0.1, 0.25, and 0.5 mg/mL) for 24 h were plated in the upper chamber and the number of cells moved to the underside of the coated membrane were counted 6 h later under light microscopy. The results showed that the number of cells invaded that the lower chamber were significantly decreased with the 24-h treatments of HLE. Such a reduction was concentration-dependent with a 60% decrease (*p* < 0.01) when HLE was at 0.5 mg/mL (Figure 2C).

### 3.3. Effects of HLE on the MMPs Activities and Expressions of LNCaP Cells

**Figure 3:**
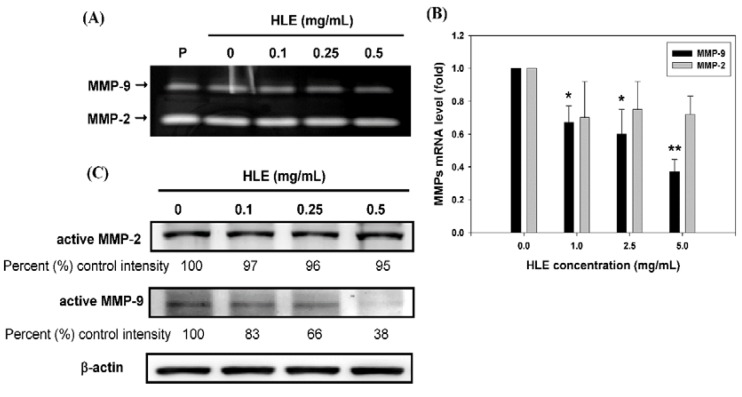
Effects of *Hibiscus sabdariffa* leaf extract (HLE) on the MMPs activities and expressions. (**A**) LNCaP cells in serum-free medium were treated with various concentrations (0, 0.1, 0.25, and 0.5 mg/mL) of HLE for 24 h. The culture medium of cells after treatment was subjected to gelatin-zymography to analyze the activity of MMP-2/9. (**B**) Real-time quantitative RT-PCR of mRNA levels and (**C**) Western blot analysis of protein levels of MMP-2 and MMP-9 in cells treated with various concentrations of HLE and harvested at 24 h. β-actin was served as an internal control of protein level. Determined expression of the protein was subsequently quantified by densitometric analysis with that of control being 100% as shown just below the gel data. The quantitative data were presented as means ± SD of three repeats from three independent studies. * *p* < 0.05, ** *p* < 0.01 compared with the control.

Because ECM degradation is crucial to cellular invasion, indicating the inevitable involvement of matrix-degrading proteinases [6,7,8,35], the effect of HLE on MMPs activities was investigated by gelatin-zymography under a condition of serum starvation to clarify the contribution of MMPs in the inhibitory effect of HLE on the invasion ability of cells. As shown in Figure 3A, MMP-9 activity was tremendously reduced by HLE in a concentration-dependent manner, whereas MMP-2 activity was little-affected. In order to confirm further the down-regulatory effects of HLE on MMP-9, quantitative real-time PCR analysis was performed. It was revealed that the treatment of 0.5 mg/mL of HLE for 24 h significantly reduced the mRNA level of MMP-9 to about 37% control intensity, and MMP-2 did not show any significant decrease (Figure 3B). The HLE-mediated change in the mRNA level of MMP-9 coincided well with the protein level as evidenced by Western blot results (Figure 3C), indicating that HLE might regulate MMP-9 expression at the transcriptional level.

### 3.4. Effects of HLE on the NF-κB Nuclear Translocation Level of LNCaP Cells

**Figure 4:**
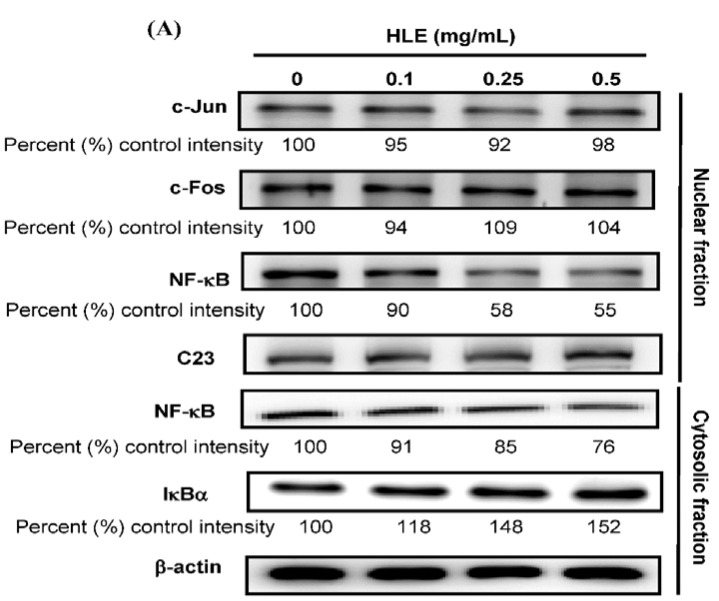

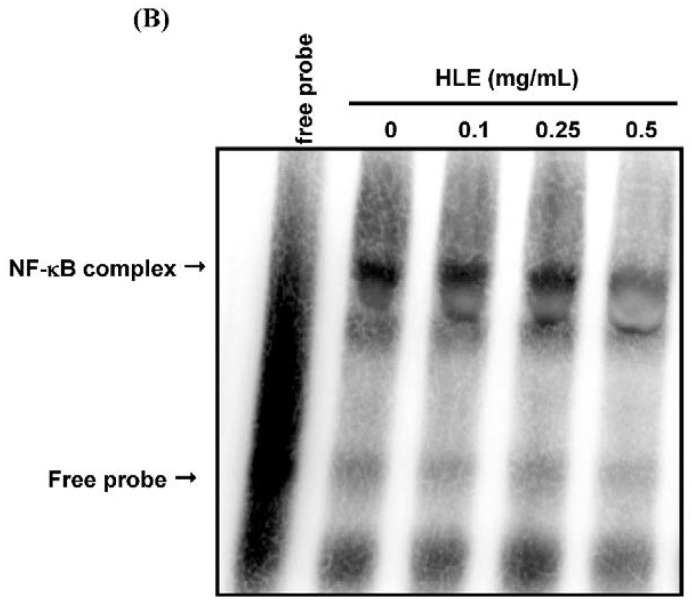
Effects of *Hibiscus sabdariffa* leaf extract (HLE) on the NF-κB nuclear translocation level. LNCaP cells in serum-free medium were treated with various concentrations (0, 0.1, 0.25, and 0.5 mg/mL) of HLE for 24 h. (**A**) The nuclear levels of c-Jun, c-Fos, and NF-κB in the nuclear fraction and the cytosolic level of NF-κB and IκBα in the cytosolic fraction were determined by Western blotting. β-actin and nucleolin (C23) were respectively served as an internal control of cytosolic and nuclear fractions. Determined expression of the protein was subsequently quantified by densitometric analysis with that of control being 100% as shown just below the gel data. (**B**) The nuclear extracts were analyzed for NF-κB DNA-binding activity using the biotin-labeled NF-κB specific oligonucleotide by EMSA. Lane 1 represented nuclear extracts incubated with unlabeled oligonucleotide (free probe) to confirm the specificity of binding.

Previous reports have demonstrated that MMP-9 promoter contains several transcription-factor-binding elements, including binding sites for AP-1 and NF-κB [6]. Therefore, the signal transduction pathway of AP-1 and NF-κB may play important roles in the regulation of MMP-9 expression. We tested next whether HLE perturbed the translocation of AP-1 and NF-κB into the nucleus of LNCaP cells by immunoblotting analysis of the nucleus extracts prepared from the treated cells. The data in Figure 4A demonstrated that the nuclear level of NF-κB (p65) was decreased to 55%–58% of control intensity after the 24-h treatments of HLE at 0.25–0.5 mg/mL, while no noticeable change was observed in the nuclear translocation of c-Jun and c-Fos (components of transcription factor AP-1). Furthermore, not only a marked reduction of nuclear NF-κB level (line 3) was found, but also a coincided decrease in the cytosolic level of NF-κB (line 5) concomitantly with an increase in the amount of cytosolic IκBα protein (line 6) was observed upon the treatments of HLE (Figure 4A). EMSA analysis also confirmed a decrease in the DNA-binding activity of the nuclear translocated NF-κB, but not AP-1 (data not shown), in the cells treated with HLE for 24 h (Figure 4B). It is therefore possible that the inhibitory effect of HLE on the motility and invasion of LNCaP cells was conducted via inactivating NF-κB that subsequently led to a reduction in MMP-9 expression.

### 3.5. Effects of HLE on the PI3K/Akt Signaling in LNCaP Cells

**Figure 5:**
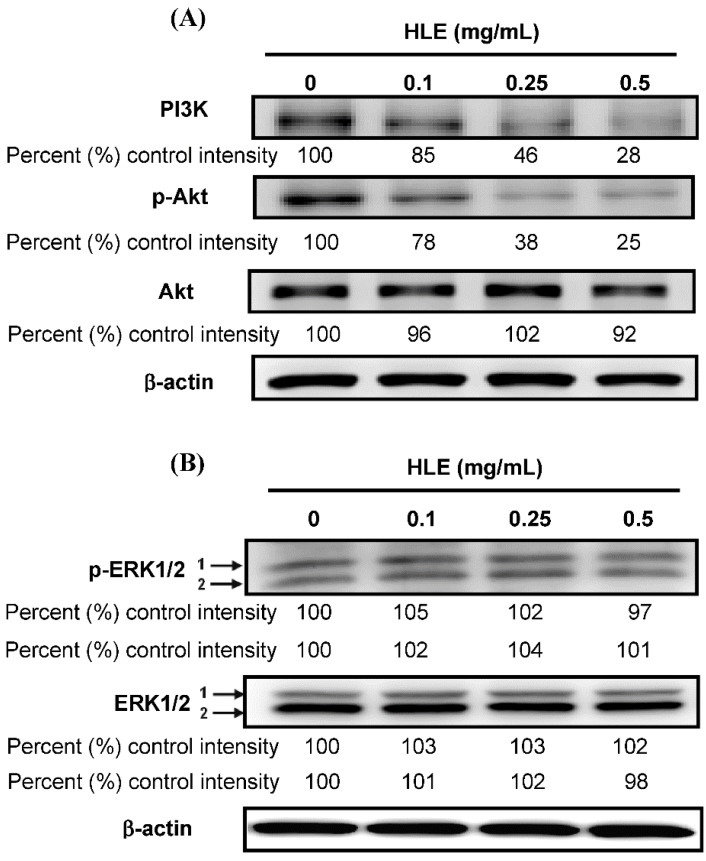
Effects of *Hibiscus sabdariffa* leaf extract (HLE) on the expressions of PI3K/Akt and ERK. LNCaP cells were treated with various concentrations (0, 0.1, 0.25, and 0.5 mg/mL) of HLE for 24 h. The cellular levels of PI3K, p-Akt, and Akt (**A**); p-ERK1/2 and ERK1/2 (**B**) were analyzed by Western blotting. β-actin served as an internal control of protein level. Determined expression of the protein was subsequently quantified by densitometric analysis with that of the control being 100% as shown just below the gel data.

Recent studies reported that the extracellular signal-regulated kinase (ERK) MAPK and PI3K/Akt signaling is involved in CaP cell migration and metastasis [33,37]. To further investigate the involvement of PI3K/Akt and ERK1/2, a series of studies was performed in which the expression of candidate signaling molecules was measured upon HLE stimulation. Incubation of LNCaP cells with HLE (24 h) led to a dose-dependent inhibition of PI3K and phospho-Akt expression (Figure 5A), while no effect was found on ERK1/2 (Figure 5B). Phosphorylated Akt (Ser473) could be suppressed after 0.1 mg/mL of HLE addition and remained reduced to 0.5 mg/mL at about 25% of control intensity (*p* < 0.01). As shown in Figure 5A, the 24-h treatments of HLE inhibited the expression of PI3K and phospho-Akt at a concentration ranging from 0.1 to 0.5 mg/mL, a dose span that coincided with the kinetics of cell migration and invasion (Figure 2B,C).

### 3.6. Effects of Mutant Akt Expression Vector on HLE-Mediated Cellular Events

**Figure 6:**
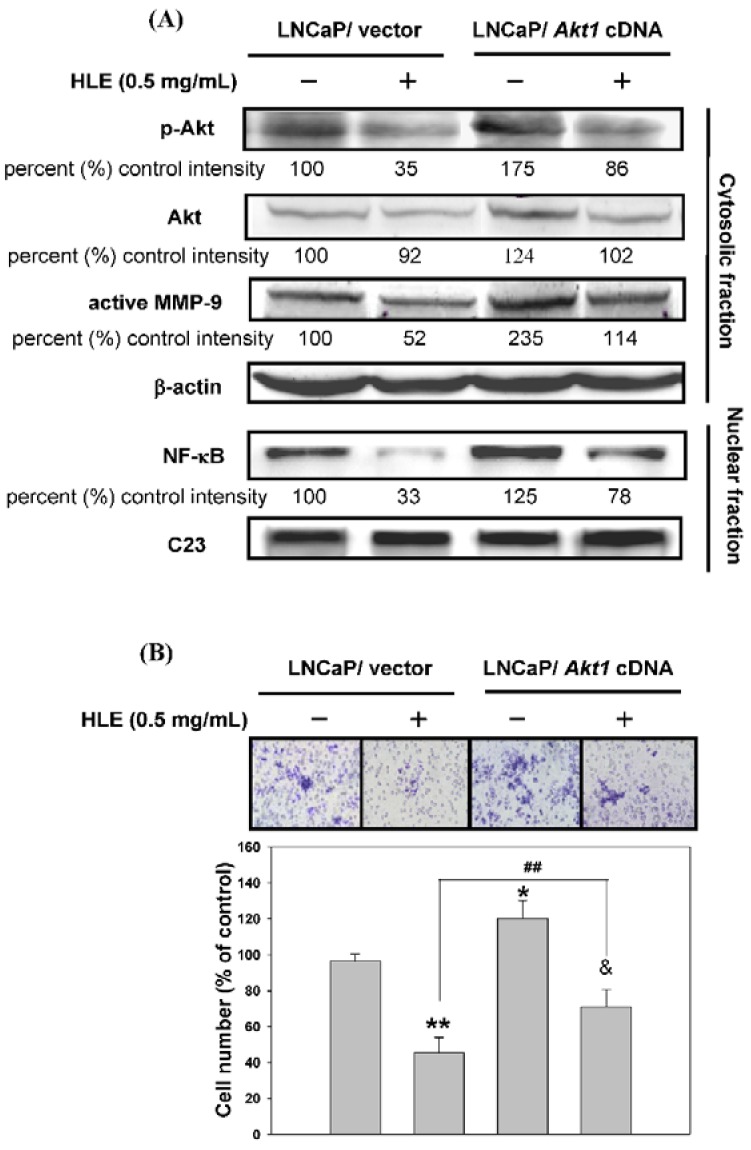
Effects of mutant Akt expression vector on *Hibiscus sabdariffa* leaf extract (HLE)-regulated cell invasion-related molecular proteins. LNCaP cells were transfected with empty vector or *Akt1* cDNA (activated) and treated with or without 0.5 mg/mL of HLE for 24 h. (**A**) The cellular levels of p-Akt, Akt, MMP-9 (upper panel), and nuclear NF-κB (bottom panel) were analyzed by Western blotting. β-actin and C23 were respectively served as an internal control of cytosolic and nuclear fractions. Determined expression of the protein was subsequently quantified by densitometric analysis with that of the control being 100% as shown just below the gel data. (**B**) Cell invasion was analyzed by Boyden chamber assay. Representative photomicrographs of the membrane-associated cells were assayed by Giemsa stain. The purple parts indicated the membrane-associated cells. “% of control” denotes the mean number of cells in the membrane expressed as a proportion of that untreated LNCaP/vector group and the average of three independent experiments ±SD. * *p* < 0.05, ** *p* < 0.01 compared with untreated LNCaP/vector group. ^&^ *p* < 0.05 compared with untreated LNCaP/*Akt1* cDNA group. ## *p* < 0.01 compared with HLE-treated LNCaP/vector group.

In this study, we had found that HLE could inhibit cell migration/invasion, down-regulate PI3K/Akt, and reduce MMP-9 expression in LNCaP cells. In order to confirm the role of Akt in HLE-mediated cellular events, we used a genetic approach to over-express Akt in LNCaP cells. The Western blotting results showed that the cells expressed as a control vector indeed had diminished levels of phospho-Akt and MMP-9 when cells were treated with HLE (lanes 1 and 2, Figure 6A). This suppressive effect of HLE was reversed by an atypical over-expression of Akt (lanes 2 and 4, Figure 6A). The expression of constitutively active Akt also improved the invasion ability of LNCaP cells that were originally inhibited by HLE as analyzed by transwell assay (lanes 3 and 4, Figure 6B).

### 3.7. Effect of HLE on Tumor Growth and PI3K/Akt/MMP-9 Signaling Pathway in the Xenograft Model

**Figure 7:**
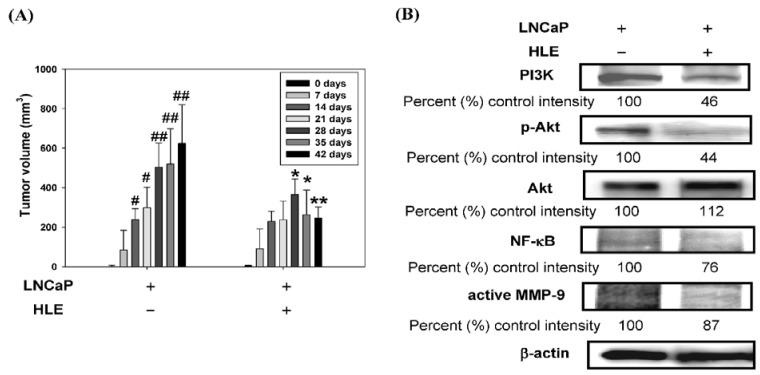

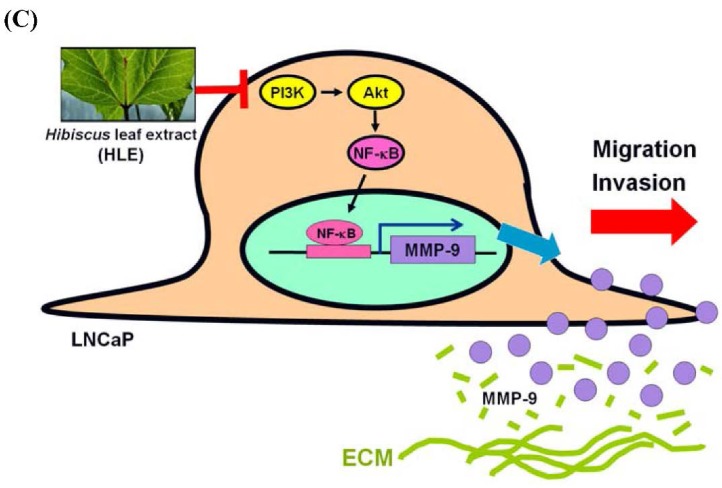
Effect of *Hibiscus sabdariffa* leaf extract (HLE) on LNCaP tumor growth in the xenograft model. LNCaP cells were injected subcutaneously into nude mice. At the same time, one of the groups was orally treated with HLE (1.0%). The mice were sacrificed after 42 days, and tumors were collected for analysis. (**A**) Tumor volume of athymic nude mice at indicative day after tumor inoculation. The values are represented as mean ± SD. ^#^ *p* < 0.05, ^##^ *p* < 0.01 compared with day 0 of the LNCaP alone group. * *p* < 0.05, ** *p* < 0.01 compared with the respective time point of the LNCaP alone group. (**B**) Western blot analysis of PI3K, p-Akt, Akt, NF-κB, and active MMP-9 protein expressions was carried out with tumor tissue extracts. β-actin was served as an internal control of protein level. Determined expression of the protein was subsequently quantified by densitometric analysis with that of control being 100% as shown just below the gel data. (**C**) A proposed model for the HLE-mediated inhibition of human prostate cancer cell migration and invasion.

To further evaluate the anti-tumor effect of HLE, an *in vivo* anti-tumor study using a nude mice xenograft model by a subcutaneous inoculation of LNCaP cells was performed. The tumor sections were used for volume analysis. Small solid tumors were observed 14 days following cell inoculation and a 50% reduction of tumor volume by HLE (1.0%) feeding was seen on day 42, compared to control animals (Figure 7A). The data indicate that HLE significantly suppresses prostate tumor growth *in vivo*, without any apparent signs of toxicity of HLE by diet consumption and body weight monitoring throughout the experiment. Furthermore, the metastasis-related proteins were expressed strongly in the tumor inoculated by LNCaP cells as determined by Western blotting (Figure 7B). HLE down-regulated the expressions of PI3K, p-Akt, NF-êB, and MMP-9 to 46% ± 9.1%, 44% ± 9.3%, 76% ± 10.0%, and 87% ± 3.3% of the LNCaP alone group (*p* < 0.01), which were suggested to be involved in the HLE-inhibited metastasis in the cell model and were indeed reduced in the specimens obtained from the HLE-treated animals.

## 4. Discussion

Nutri-medicine is based on the premise that plants contain natural nutrients that can promote health and alleviate illness [38]. Nutri-medicinal plants refer to not only herbaceous plants, but also to roots, bark, leaves, flowers, and seeds. The calyces of *Hibiscus* flowers are used to prepare hot (sour tea) and cold beverages that are consumed worldwide. Due to their perceived potential health benefits, commercial preparations of *H. sabdariffa* extracts (HSE) are currently marketed as supplements and these extracts have gained an important position in some local soft drink markets [18]. In folk herbal medicine, it is used to treat hypertension [12,13], pyrexia [12], and liver disorders [14,15] and it is also taken for its immunemodulating effects [16] The leaf of the plant is usually discarded around the world, except in Africa, where it is consumed as a vegetable. An ethanol extract of the dried leaves has been shown to reduce aflatoxin formation [39], and to have an *in vitro* inhibitory effect against some fungi that include *Aspergillus fumigatus*, *Rhizopus nigricans*, and *Trichophyton mentagrophytes* [40]. Studies with laboratoryanimals have demonstrated that an aqueous extract of *H. sabdariffa* leaves (HLE) caused a significant decrease in epididymal sperm counts, histological distortion of tubules, disruption of normal testicular epithelial organization, and disintegration of sperm cells. The authors postulated that these effects were related to interference of spermatogenesis by the extract, which may have been caused by an estrogenic-like action of the extract [23]. Furthermore, many investigations have highlighted that HLE may act as an antioxidant, antihyperlipidemic, and anticancer agent [20,21,27]. Based on the existing information regarding the anticancer effect of *H. sabdariffa* leaf from *in vitro* and *in vivo* studies, very little information is available about the influence of HLE on cancer cell metastasis and invasion. To our knowledge, this is the first report showing the anti-invasive activity of *H. sabdariffa* leaf.

To demonstrate HLE as a regulator of cell migration and invasion, we excluded the effect of HLE on tumor cell growth by MTT assay, showing that the cell viability was not significantly altered by the treatment of HLE at concentrations less than 0.5 mg/mL (Figure 2A). Many traditional herbs have been reported to exert differential functions in different cell types under different concentrations [41,42]. As our study showed, HLE at lower concentrations (0.1–0.5 mg/mL) inhibited cell migration and invasion via a sustained inactivation of the PI3K/Akt signal in LNCaP cells (Figure 2, Figure 5 and Figure 6), and, alternately, a higher dosage (2.5 mg/mL) induced apoptosis via both intrinsic (Bax/cytochrome c-mediated caspase 9) and extrinsic (Fas-mediated caspase 8/t-Bid) apoptotic pathways [27]. Our findings, hence, provided evidence supporting a multifunction of HLE on LNCaP cells between high and low concentrations. HLE at a concentration ranging from 0.1 to 0.5 mg/mL was applied in all subsequent experiments to avoid the influence of cell growth on the observed parameters. HLE treatment was shown to inhibit both the migration and invasion potential of LNCaP cells by wound-healing analysis and *in vitro* transwell (Figure 2B,C). Our findings highlight that HLE plays an inhibitory role in malignant progression and provides an additional pathway for its anticancer activity.

An increased expression of MMPs has been shown to be related to an invasive phenotype of cancer cells. It is also of paramount importance to note that expressions of MMP-2 and MMP-9 are associated with CaP progression [43]. The inhibition of MMP-2 and MMP-9 expression suppresses the metastatic potential of CaP [44]. The present study demonstrated that HLE inhibited the secretion of MMP-9, but not MMP-2, in LNCaP cells (Figure 3A). HLE had an effect not only on the activity of MMP-9, but also on the mRNA and protein levels (Figure 3B,C). The transcription of the MMP-9 gene is regulated by upstream regulatory elements including NF-κB and AP-1 binding sites [6]. Indeed, one or more of these binding sites have been implicated in mediating the effects of a diverse set of agents. Consequently, suppression of the activity of NF-κB, c-Fos and c-Jun, or blockage of their binding to respective regulatory elements, potentially inhibited tumor invasion [35,45]. Consistent with these findings, the treatments of HLE to LNCaP cells repressed NF-κB DNA-binding activity (Figure 4B), which was accompanied by a decrease in nuclear translocation of the factor (Figure 4A). It is therefore possible that the inhibitory effect of HLE on the motility and invasion of LNCaP cells was via the inactivation of NF-κB, and afterward, MMP-9.

Multiple genetic changes take place during the process of carcinogenesis. Identifying key proteins, such as PI3K, Akt, and ERK MAPK involved in these processes is vital for understanding carcinogenesis and devising new therapies. The PI3K/Akt signaling pathway is implicated in cell migration and invasion [33,35]. Recent studies showed that the signaling pathway is important in the metastasis of CaP cells [46]. In order to test if HLE targets the PI3K/Akt signaling-mediated cellular events in LNCaP cells, the effect of HLE on these signaling molecules was investigated by immunoblotting. In agreement with these reports, we observed that HLE caused a dose-dependent decrease in cellular levels of PI3K and phosphorylated Akt (Figure 5A). However, there was no noticeable change in cellular and phosphorylatory levels of ERK1/2 within the same periods (Figure 5B). Additionally, it was reported that the MMP-9 mRNA level was correlated with the PI3K/Akt/mammalian target of the rapamycin (mTOR) pathway [47]. mTOR, one of the downstream targets of Akt, was one mechanism in a tightly regulated network of intracellular signal pathways and contributed to cell invasion [48]. Thus, to identify whether the mTOR was related to HLE-inhibited MMP-9, further work is needed. In addition to its role in controlling cancer development, mTOR also contributes to hyperglycemia and hyperlipidmia [49,50]. In this context, the anti-diabetic drug metformin, which is known to negatively regulate the action of mTOR, improves metabolic profile and represents another interesting approach to improve insulin sensitivity [51]. Consistent with this idea, we asked whether down-regulation of the PI3K/Akt/mTOR signaling pathway, in turn, might contribute to the hypoglycemic and hypolipidemic effects of HLE. However, their relevance needs to be demonstrated.

Through our experiments, a new link emerges between MMP-9 expression and levels of phosphorylated Akt, which alters both cell migration and cell invasion. LNCaP cells transfected with *Akt1* cDNA (activated) showed an increase in phosphorylated Akt and increases in MMP-9 and nuclear NF-κB expressions (Figure 6A). Overexpression of Akt activity also resulted in an increase in migration, measured in a wound-induced migration assay (data not shown) and in cell invasion in a transwell assay (Figure 6B). Furthermore, the PI3K/Akt signaling pathway induces the expression of NF-κB transcription factor [52]. Our genetic evidence supported these literatures and cooperatively demonstrated that PI3K/Akt signaling played a crucial role in HLE-inhibited migration/invasion in the current study. Because of its critical role as an antagonist of PI3K/Akt/NF-κB, HLE might, in turn, have a significant impact on the mechanism that inhibits the MMP-mediated cellular events in CaP cells.

To determine whether the dose of HLE found inhibitory in the nude mice bears any relation to those used clinically in humans, an interspecies scaling factor of 12.3 was applied to generate mouse-equivalent doses [53,54]. This factor reflects the 12.3-fold difference in surface area-to-body weight ratio between mice (0.0066 m^2^/0.02 kg) and humans (1.6 m^2^/60 kg); consequently, 12.3 times more extract is required in the mouse to be comparable to the dose in humans. Thus, in the present study, the dose of HLE, used at 1% in mice, might be useful in determining approximate dosing ranges for effective use in humans. Furthermore, many used concentration of plant-derived polyphenols are being evaluated in the relevance for those achieved in the human body. These findings implied the possibility that differences in experimental design, including anti-oxidative DNA damage, anti-lipid peroxidation, anti-atherosclerosis, and hepatoprotection effects, could be subjected to 0.5–10% concentrations of plant-derived polyphenols has been shown in Table S1. Several of the studies establishing the potential of HLE were carried out in animals. However, the exact dose of HLE to be used in the human body to moderately inhibit cancer cell invasion and metastasis has not yet been examined in full detail. In order to obtain a safe dose for HLE to inhibit tumor metastasis, more clinical trials need to be done.

## 5. Conclusions

To summarize, we proposed a schematic presentation of possible mechanisms for the inhibitory effect of HLE on migration and invasion of LNCaP cells (Figure 7C). HLE treatments inhibited the cell invasion via down-regulation of PI3K/Akt signaling and further inactivation of NF-κB, followed by a reduction of MMP-9 expression. Most significantly, HLE inhibited the growth of prostate tumor xenograft in athymic nude mice (Figure 7A). Our data also showed that HLE down-regulated the Akt/NF-κB/MMP-9 signaling pathway *in vitro* and *in vivo* (Figure 7B). HLE represents an accessible possible source of polyphenolic compounds useful for the preparation of food supplements. Our findings indicate that HLE could be developed as potent anticancer agent and used in natural, healthy foods for the management of CaP. The global characteristics of *H. sabdariffa* leaf suggest that it could have important agricultural applications, adding to the value for the culture of this nutri-medicine plant.

## Supplementary Materials

Supplemental Table S1—Comparison of various plant-derived polyphenols. Supplemental Figure S1—Effects of ellagic acid (EA) and *Hibiscus sabdariffa* leave extract (HLE) on pH value under the culture conditions.
