# Supplementary material for: Hibiscus sabdariffa Leaf Extract Inhibits Human Prostate Cancer Cell Invasion via Down-Regulation of Akt/NF-κB/MMP-9 Pathway
Source: Nutrients. 2015 Jun 24;7(7):5065–87. doi: 10.3390/nu7075065 (PMC4516987; doi:10.3390/nu7075065)
Supplement: Supplementary file 1 [file nutrients-07-05065-s001.docx]

**Supplementary Information**

**Table S1.** Comparison of various plant-derived polyphenols.

|  | | **HLE** ^a^ | **HSE** ^b^ | **GTE** ^c^ | |  |
| --- | --- | --- | --- | --- | --- | --- |
| Full name | | aqueous extracts from the leaves of *Hibiscus* *sabdariffa* | aqueous extracts from the flower of  *Hibiscus* *sabdariffa* | Green tea extract | |  |
| Component | | anthocyanins, flavonoids,  phenolic acids | anthocyanins, flavonoids, phenolic acids | EGCG ^d^, EGC ^e^, ECG ^f^, EC ^g^, caffeine | |  |
| Major compound (%) | | EA ^h^ (33.6%) | N/A ^i^ | EGCG (30%–42%) | |  |
| Anti-cancer and oxidative DNA damage | Animal models | 1% (athymic nude mice) | 1–2% (ICR mice ^j^) | 2% (A/J mice) | |  |
| Anti-lipid peroxidation and atherosclerosis | Human studies | N/A | N/A | | 3 g/day; 10% | |
|  | Animal models | 300 mg/kg (Wistar rats) | 0.5%–1% (New Zealand White rabbits) | | 50 mg/kg; 3% (Wistar rats) | |
|  | Human studies | N/A | 1 g/day | | 600 mg/day | |
| Hepatoprotection | Animal models | N/A | 1%–5% (Wistar rats); 200–600 mg/kg (BALB/c mice) | 2.5% (Wistar rats) | |  |
| Hypoglycemic | Human studies | N/A | N/A | | N/A | |
|  | Animal models | 250 mg/kg (Wistar rats) | 200 mg/kg (SD rats ^k^) | | 500 mg/kg (SD rats) | |
|  | Human studies | 1 mg/day | 2 g/day | | 857 mg/day | |
| References | | Kuriyan *et al.*, 2010; Ochani & D’Mello, 2009; Sachdewa *et al.*, 2001; Lin *et al.*, 2012 | Chen *et al.*, 2005; Lin *et al.*, 2007; Liu *et al.*, 2006; Liu *et al.*, 2010; Mozaffari-Khosravi  *et al.*, 2008; Pheng *et al.*, 2011 | Higdon & Frei, 2003; Hsu *et al.*, 2011; Kuzu *et al.*, 2008; Lin *et al.*, 1998; Li *et al.*, 1999; Miura *et al.*, 2000; Sano *et al.*, 1995; Xu *et al.*, 1992 | |  |

^a^ HLE, *Hibiscus sabdariffa* leaf extract; ^b^ HSE, aqueous extract of dried flowers of *H. sabdariffa*; ^c^ GTE, green tea extract; ^d^ EGCG, (-)-epigallocatechin gallate; ^e^ EGC, (-)-epigallocatechin; ^f^ ECG, (-)-epicatechin gallate; ^g^ EC, (-)-epicatechin; ^h^ EA, ellagic acid; ^i^ N/A, not available; ^j^ ICR mice, Institute of Cancer Research mice; ^k^ SD rats, Sprague Dawley rats.


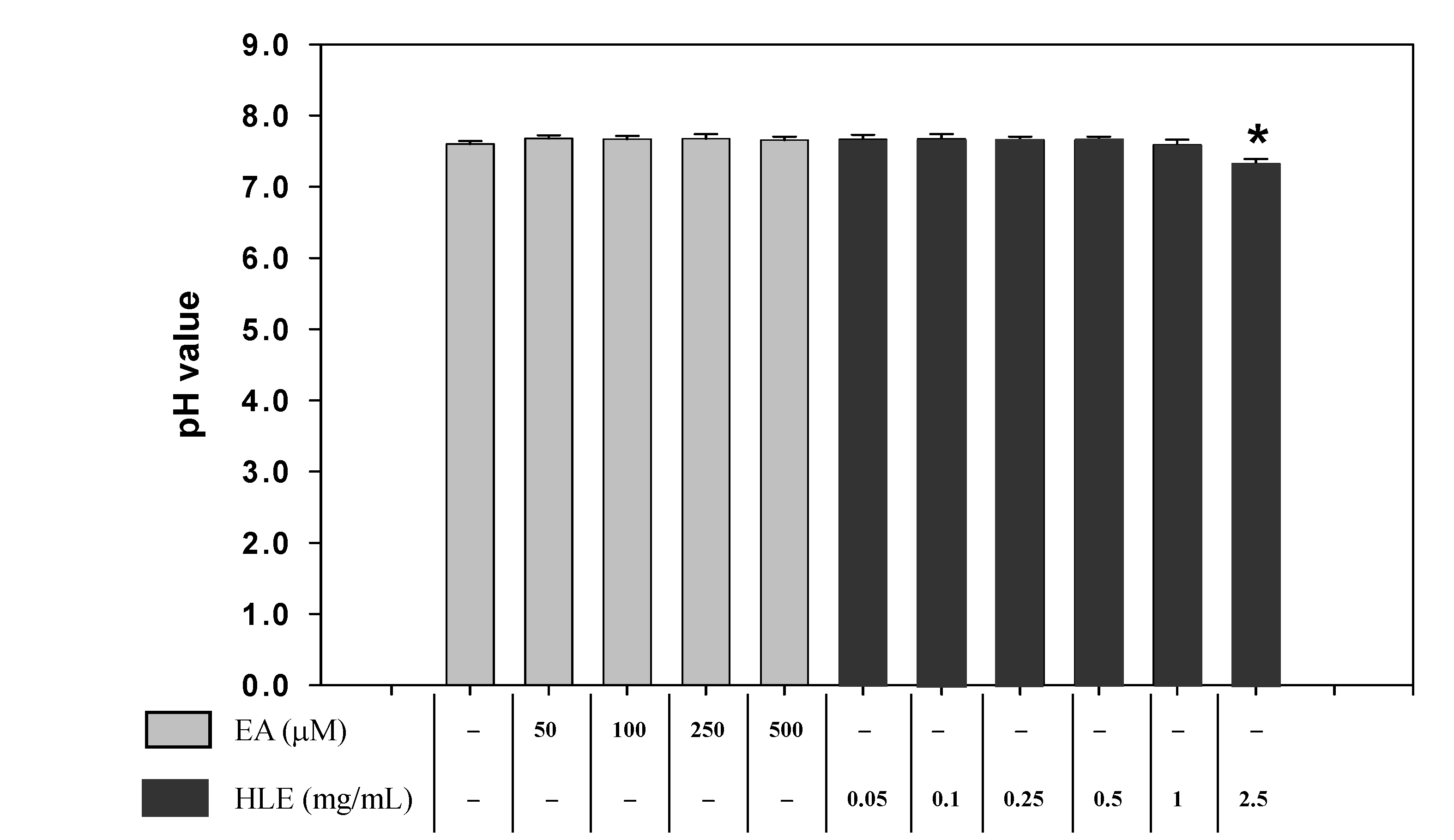


**Figure S1.** Effects of ellagic acid (EA) and *Hibiscus sabdariffa* leave extract (HLE) on pH value under the culture conditions. LNCaP cells were treated with or without EA or HLE under different concentrations. The pH value of the culture medium was analyzed by pH meter. The result represents the average of three independent experiments ±SD. * *p* < 0.05 compared with control.

© 2015 by the authors; licensee MDPI, Basel, Switzerland. This article is an open access article distributed under the terms and conditions of the Creative Commons Attribution license (http://creativecommons.org/licenses/by/4.0/).
